# Vinorelbine as First-Line Treatment in Stage IV Canine Primary Pulmonary Carcinoma

**DOI:** 10.3390/vetsci10120664

**Published:** 2023-11-22

**Authors:** Valentina Rinaldi, Riccardo Finotello, Andrea Boari, Emanuele Cabibbo, Paolo Emidio Crisi

**Affiliations:** 1 Department of Veterinary Medicine, University of Teramo, 64100 Teramo, Italy; aboari@unite.it (A.B.);; 2 Polo Oncologico Veterinario, AniCura Italy Holding S.r.l., 40100 Bologna, Italy; 3 VetCenter, VetPartners, 43121 Parma, Italy

**Keywords:** dog, lung, vinorelbine, staging, chemotherapy

## Abstract

**Simple Summary:**

Primary lung tumours are relatively rare in dogs but when they present, surgical excision, with or without the use of chemotherapy, represents the first-line treatment option; however, when they present as advanced inoperable disease, options are limited. Vinorelbine (VRL) is a chemotherapy drug that has shown to reach 300-fold higher concentrations in the lungs compared to plasma, has proven anticancer activity and it has been approved for the treatment of non-small-cell lung cancer in humans. In this retrospective study, we have enrolled ten dogs with advanced primary lung cancer that were treated with vinorelbine as a first-line treatment strategy. Partial response was documented in eight dogs (80%). Median time to progression was 88 days (range: 7–112) and median survival time for all dogs was 100 days (range 7–635). VRL was well tolerated with an adequate toxicity profile and provided partial responses effective in the treatment of inoperable canine lung tumours.

**Abstract:**

Vinorelbine (VRL), a semi-synthetic vinca alkaloid commonly used in humans with advanced lung cancer, reaches high concentrations in the lung tissue, has proven antineoplastic activity and a low toxicity profile in dogs. Treatment-naïve, client-owned dogs with a cyto/histological diagnosis of advanced pulmonary carcinoma, selected from a laboratory database and previously subjected to imaging, were enrolled in the study. Vinorelbine (15 mg/m^2^) was administered weekly for 4 weeks and then fortnightly until progressive disease was documented. Staging work-up was repeated by means of diagnostic imaging after the fourth VRL (i.e., 28 days) and monthly thereafter; response to treatment was evaluated according to the RECIST. Toxicity was graded following the VCOGC group. Ten dogs met the inclusion criteria. Partial response was documented in eight dogs. Median time to progression was 88 days (range: 7–112) and median survival time for all dogs was 100 days (range 7–635). The most common side effect was neutropenia. The main limitations of the study were the absence of histological diagnosis in eight cases and the limited number of treated dogs. VRL is well tolerated with an adequate toxicity profile and may be useful in the management of advanced lung tumours if used as a first-line treatment strategy.

## 1. Introduction

Primary pulmonary tumours represent 1% of neoplastic disease in companion animals [1,2,3], and approximatively 85% are epithelial in origin [4,5]. Local invasion and metastatic disease, which occur via lymphatic or hematogenous dissemination, occur in 23% to 71% of the cases [2,3,5]. Tumour grade, clinical signs, histologic subtypes, and lymph node involvement are associated with survival time (ST) [6,7]. The most common clinical signs at presentation include dyspnoea, lethargy, hyporexia, weight loss and hypertrophic osteopathy; however, the diagnosis is incidental in up to 30% of the patients [8,9]. Surgical excision is the treatment of choice; however, the diagnosis is often made at advanced clinical stages when the tumour has already invaded neighbour structures and/or has metastasized, making palliative strategies a more realistic option. In a recent study [10], the effect of hypofractionated radiotherapy as neoadjuvant therapy for canine solitary lung adenocarcinoma was described. Results were encouraging in terms of tumour response; although acute and late radiation-induced toxicity were common, it was manageable with short-term anti-inflammatory treatment. Different chemotherapeutics have been used in the treatment of canine lung tumours, such as platinum compounds, vinca alkaloids and anthracyclines [9,11,12], but no medical treatment has been yet defined as the gold standard. Vinorelbine (5′-noranhydrovinblastine; VRL) is a semi-synthetic vinca alkaloid that exerts cytostatic activity on tumour cells [13,14]. VRL induces metaphase arrest in dividing cells, disrupting the microtubules [14], and it has been approved for the treatment of non-small-cell lung cancer (NSCLC) in humans, either as a single agent or in polychemotherapy regimens [15]. Clinical pharmacokinetic data showed that VRL concentration in the human lungs was 300-fold higher compared to plasma [16], which is thought to be also the case in small animal patients, although the data have not been confirmed. In veterinary medicine, the pharmacokinetics of VRL was investigated following intravenous (IV) administration to mice, rats and dogs at a dose of 0,4 mg/Kg [17]. In a clinical study with 19 dogs affected by different neoplasia, a dosage of 15 mg/m^2^ IV administered weekly for four treatments, followed by four treatments 2 weeks apart, has been considered safe [12]. In this population, there had been a 28,5% response rate. As with other vinca alkaloids, the adverse event profile of vinorelbine includes myelosuppression, which is manifested predominantly as neutropenia, and gastrointestinal disturbances such as nausea, vomiting, diarrhoea and constipation [14]. The use of VRL in canine neoplasia is still infrequent and only a limited number of studies have been published to date [12,18,19,20]; a summary of the adverse events is reported in Table S1.

The staging system commonly used for canine pulmonary tumours was established in 1980 [21] (Table 1), but in a recent publication [22], authors have instead proposed the use of a human-derived lung cancer classification system [23] that combines the clinical stage with the TNM system (Table 2). Lee et al. [22] have highlighted that Owen’s classification has not been updated to reflect the advancement of diagnostic imaging and how this is not clear enough or prognostically intuitive [21]. Conversely, the modified classification [22,23], designed to be a clearer and easier method of communicating local and metastatic tumour extent, proved to have prognostic significance and to better reflect the severity of tumour burden [22]. The aim of this study was to retrospectively investigate treatment with VRL in dogs with lung carcinoma stage IV [23] and to define anti-tumour response and toxicity in these patients.

## 2. Materials and Methods

### 2.1. Study Population

Medical records of the Veterinary Teaching Hospital, Department of Veterinary Medicine of the University of Teramo (Italy) and at the Clinica Veterinaria Jenner, VetPartners, Parma (Italy), were retrospectively searched between January 2018 and December 2022. Dogs with a cytological or histological diagnosis of pulmonary carcinoma in clinical stage IV, according to the human classification scheme previously adapted to dogs [23], were considered eligible. To be included in the study, dogs had to be treatment-*naïve* and clinical data had to include a complete physical examination, complete blood count (CBC), serum biochemistry, thoracic radiographs and abdominal ultrasound (US) or computerised tomography (CT) performed prior to any oncological treatment (i.e., surgery and/or chemotherapy).

### 2.2. Treatment

Vinorelbine was administered once a week for four weeks and then every other week (EOW) until progressive disease was documented (PD) [19]. Vinorelbine, diluted in saline solution (NaCl 0.9%), was injected by means of an indwelling catheter placed in a peripheral vein for no less than 10 min. Vinorelbine toxicity was assessed via owners’ verbal reports, physical examination and/or clinical pathologic data and classified according to the Veterinary Cooperative Oncology Group (VCOG) [24]. Physical examination and CBC were performed prior to VRL administration and treatment was delayed when the neutrophil count was lower than 1500 cells/μL; chemotherapy nadir was not reassessed nor routinely checked in the EOW part of the protocol.

### 2.3. Follow-Up

Thoracic radiography and abdominal US were planned once a month after starting the chemotherapy protocol to monitor for tumour’s response. Measurements of the primary tumour and target lesions were compared with those obtained at baseline. Tumour response was evaluated according to the response evaluation criteria for solid tumours in dogs [25]. However, severe deterioration/recurrence of presenting respiratory signs and/or appearance of new respiratory signs was interpreted as PD, even in the absence of repeated imaging studies. Imaging studies were interpreted by a board-certified radiologist or by a veterinarian with extensive experience in the field of radiology.

### 2.4. Endpoint

Time to progression (TTP) was calculated from the start of treatment to PD and median survival time (MST) was calculated from the start of treatment to death.

### 2.5. Statistical Analysis

Statistical analysis was performed using GraphPad Prism 6.01 software (GraphPad Software, Inc., San Diego, CA, USA). Data were evaluated using a standard descriptive statistic and reported as the mean and SD, or as median and range based on their distribution assessed using the D’Agostino Pearson test. Kaplan–Meier survival curve was used to evaluate the median survival times.

## 3. Results

### 3.1. Patient Population

Dogs’ characteristics are summarized in Table 3. Ten dogs met the inclusion criteria and were included in the study. There were four crossbreeds, two Border Collies, two English Setters, one Bernese Mountain Dog and one American Staffordshire terrier. Six dogs were male (three neutered) and four female (three spayed), they had a median age of 8 years (range 6–13) and a median body weight of 25 Kg (range 4–37). The two Border Collies were MDR1 wild type; the presence of MRD1 mutation was investigated as a precaution prior to the commencement of chemotherapy. Dyspnoea was observed in three dogs, weight loss in three cases, one dog was coughing, while the dogs had no detectable clinical signs. All the dyspnoeic dogs had pleural effusion, detected by means of thoracic ultrasound; cytological examination of the effusion revealed clusters of neoplastic epithelial cells and was considered consistent with carcinomatosis.

In two cases, pulmonary carcinoma was diagnosed histologically after tru-cut biopsies, while in the remaining eight cases, diagnosis was obtained cytologically through US-guided fine-needle aspiration (FNA). The descriptions of all cases are summarized in Table 4.

### 3.2. Administration of Vinorelbine and Adverse Events

In all dogs, VRL was administered at the dose of 15 mg/m^2^ and no dose reductions were performed throughout the protocol. The median number of VRL administrations was eight (minimum one, maximum ten). One dog received only one dose of VRL, dying seven days after commencing chemotherapy, while the remaining nine dogs received a minimum of eight administrations. In three of the dogs receiving multiple VRL doses, piroxicam was concurrently administered every other day (EOD) at the dose of 0.3 mg/Kg PO. The adverse events (AEs) related to VRL treatment were neutropenia, anorexia and vomiting. Afebrile grade I neutropenia was reported in two dogs, grade II in two dogs and febrile grade IV in one dog after the third administration. One dog showed anorexia grade II and two dogs showed vomiting grade I. The dog that developed grade IV febrile neutropenia required hospitalization and antibiotic treatment (ampicillin 20 mg/Kg EV q8h; enrofloxacin 10 mg/Kg q24h) and IV fluid therapy. The dog was no longer considered at risk after 48 h (resolution of fever and improvement of the neutrophil count); however, it was discharged after 5 days of hospitalisation and haematological normalization, as per the owner’s wishes and clinician preference. The following VRL dose was postponed to one week later; no further AE were recorded in this patient. When VRL was administered EOW, CBC was only performed in the presence of treatment-related clinical signs, which were not recorded in any of the dogs. Data were summarized in Table 4. None of the dogs received any other medication other than antineoplastic therapy (including COX inhibitors).

### 3.3. Tumour Response

**Figure 1:**
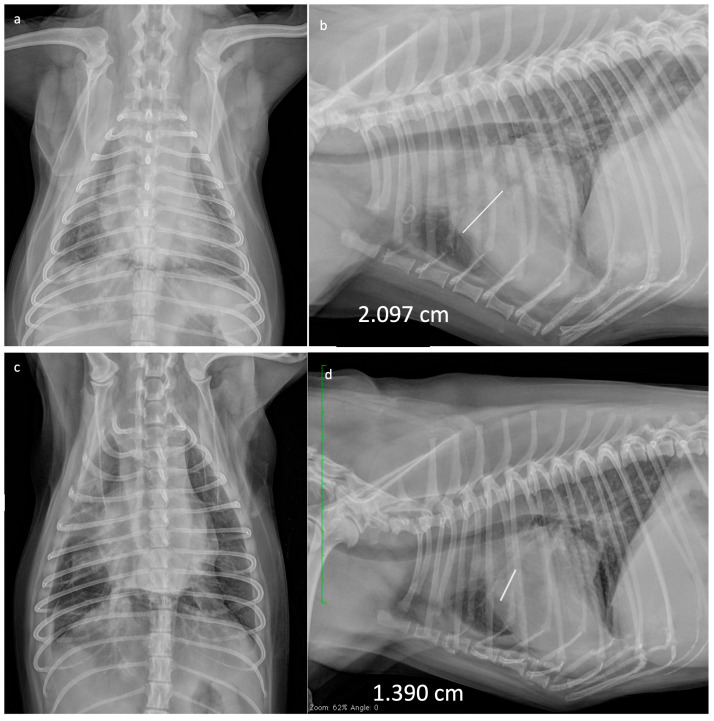
Thoracic radiographies of a 4-year-old crossbreed dog with IV stage canine primary pulmonary carcinoma at the time of diagnosis (**a**,**b**) and in PR 28 days after commencing treatment with weekly VRL (**c**,**d**). White and green lines show the maximum length of the target lesion.

**Figure 2:**
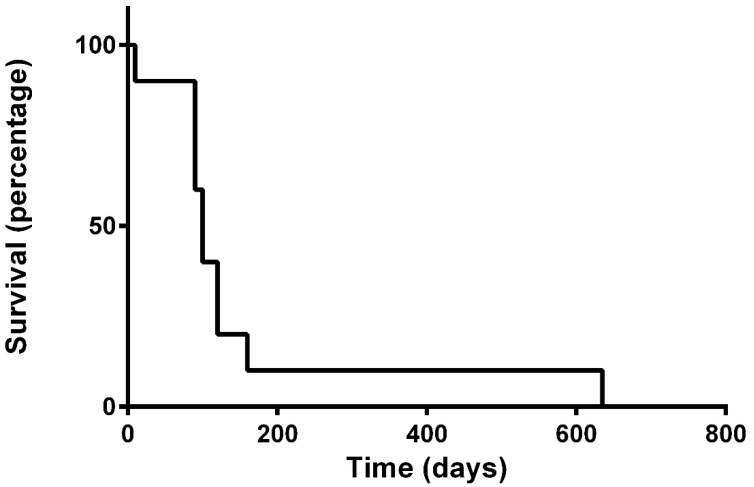
Kaplan–Meier survival curve of dogs with IV stage canine primary pulmonary carcinoma treated with vinorelbine.

In all dogs, tumour response was assessed through thoracic radiography (Figure 1). None of the dogs achieved CR. Eight dogs achieved PR (80%), one maintained SD (10%) for 56 days and one died 7 days after the first VRL (10%) due to respiratory complications; TTP was 88 days (range: 7–112). In two out of the three dogs presenting with pleural effusion, this decreased to such an extent that it no longer required therapeutic thoracocentesis after four injections of VRL; however, as pleural effusion is a non-measurable condition, this was not evaluated for tumour response. One of the dogs that initially achieved PR developed PD after 90 days from starting chemotherapy and was therefore started on rescue carboplatin (once every 21 days, for a total of six cycles), followed by metronomic chemotherapy (MC) (thalidomide 1 mg/Kg SID; cyclophosphamide 10 mg/m^2^ EOD; piroxicam 0.3 mg/Kg EOD). This patient demonstrated SD on day 545 post diagnosis, after repeated thoracic radiographs, abdominal ultrasound and blood works; PD was documented via thoracic radiographs 635 days post diagnosis, at which point euthanasia was performed. Median survival time for all dogs was 100 days (range 7–635) (Figure 2). In conclusion, nine dogs of ten were humanely euthanized due to the progressive disease, following owners’ request.

## 4. Discussion

In this study, we evaluated the antitumor response and AE of VRL when used as a first-line treatment for dogs with stage IV pulmonary carcinoma [23]. As with other vinca alkaloids, the AE profile of VRL includes myelosuppression, which is manifested predominantly as neutropenia, and gastrointestinal disturbances such as nausea, vomiting, diarrhoea and constipation [16]. In this study, the 15 mg/m^2^ dosage of VRL was well tolerated, with neutropenia being the most common AE, consistent with previous data in dogs [12,20].

Grade IV haematological AEs (neutropenia and thrombocytopenia) represent dose-limiting toxicities (DLT) in phase I clinical trials [26], and these episodes normally demand dose reductions in routine clinical scenarios also; however, decision making may vary depending on the situation. In our cohort, one case developed a single episode of grade IV febrile neutropenia following the third VRL dose, which resolved with symptomatic treatment and hospitalization. Similarly, three other dogs developed grade III neutropenia after the third dose, suggesting the potential for cumulative VRL toxicity rather than having reached the DLT or MTD, respectively. In such scenarios, there are no standardised guidelines and reducing dose density may be sufficient and even preferable to affecting dose intensity. This is particularly relevant if we consider that after the fourth VRL, dose treatments will be given fortnightly [19] to allow a longer bone marrow resting time. Ultimately, it has to be considered that developing a grade IV toxicity throughout the treatment may be a random event, or the complication of undetected comorbidities. Clinical signs observed in this cohort were comparable with those reported in the literature for dogs with advanced stage disease [7,8,9]. In detail, 30% (3/10) of our cases showed dyspnoea resulting from pleural effusion; in these cases, cytology was consistent with carcinomatosis, which is the most common cause of neoplastic effusions [27,28]. In two out of these three dogs, pleural effusion decreased to such an extent that it no longer required therapeutic thoracocentesis after four injections of VRL. This is in line with the study from Cui and colleagues [29] who reported that VRL was effective in inhibiting the formation of malignant pleural effusion in mice. Poirier et al. [12] reported that VRL was administered in seven dogs with bronchoalveolar carcinoma and, in two of these cases, there was a 50% reduction in the volume of the tumour. In another study [19], an overall TTP and ST of 55 and 92 days were reported, respectively, in 16 dogs with macroscopic disease treated with VRL as primary treatment or following other chemotherapeutic agents. In this study, VRL was administered in a small cohort of 10 dogs, resulting in a TTP and MST of 88 and 100 days, respectively, with only one dog dying after one week of therapy, while all the others showed PR after 4 weeks. These data corroborate previous observations [12,19] and would support the use of VRL as a suitable first-line treatment in dogs with advanced lung carcinoma. In humans, VRL has been evaluated as a single-agent therapy in two nonrandomized studies in NSCLC patients without previous chemotherapy, and 23 out of 69 patients (33%) demonstrated a partial response [30].

Polton and colleagues [31] reported the efficacy of MC in the treatment of advanced lung tumours, suggesting that MC has the potential to achieve a clinically equivalent and more successful outcome compared to surgery. In our study, one dog was still experiencing response one year after the diagnosis and was then switched first to carboplatin and then to MC (piroxicam, thalidomide and cyclophosphamide) at the time of PD, remaining in SD at the time of writing. The outcome of this case may suggest that even in light of advanced disease, selected patients may benefit from sequential strategies rather than only palliative approaches, such as using MC alone. COX upregulation, particularly COX-2, has been documented in human lung tumours [32] and in a variety of neoplastic diseases in animals [33]. In dogs, COX expression was demonstrated in various carcinomas [34] but has not been investigated in pulmonary carcinomas to the best of our knowledge. However, COX upregulation plays a major role in tumour-associated inflammation and angiogenesis [35] and this has mainly driven clinicians’ decision to combine VRL with piroxicam. The use of COX inhibitors alongside MTD chemotherapy or MC has therefore become a standard practice in the treatment of many diseases, such as urothelial carcinoma [36], among others. In this study, piroxicam and VRL were combined in three dogs due to clinician preference, possibly improving the clinical picture and biasing the conclusion of our study; however, we consider it unlikely that piroxicam could have driven tumour response to such an extent and we believe it is still reasonable to consider VRL as playing the major role, even in these cases.

In this study, we adopted a recent human lung cancer classification system [23], which has been previously utilised in the veterinary literature [22], demonstrating its reliable prognostic value. This strategy was preferred over the standard classification system [21] as the TNM system does not translate into clinical stages and somehow does not necessarily reflect the severity of the disease. In particular, 7/10 of our cases presented with multiple pulmonary nodules, which, according to Owen’s TNM classification, would be consistent with a T2 tumour but not with the presence of metastatic disease (M1). For this reason, we think that the refined classification better reflects the clinical scenario.

The study presents some limitations including: the limited number of treated dogs, its retrospective nature, as well as the lack of histological diagnosis for all cases and the lack of a control groups. The histologic subtype and the degree of tumour differentiation have proven to correlate with prognosis [8]; it is therefore possible that we may have included cases with a less aggressive tumour histotype, resulting in higher chances of responding to treatment and experiencing an extended survival. However, tumours were all in advanced stage, and, in the authors’ opinion, this still support an aggressive biological behaviour and somehow minimises the lack of either histological diagnosis or sub-classification.

## 5. Conclusions

Our study suggests that VRL is well tolerated with an adequate toxicity profile, and it may be useful in the management of advanced lung tumours if used as the first-line treatment strategy. However, a prospective and larger clinical trial is required to confirm the efficacy and usefulness of VRL in the management of advanced primary lung carcinomas.

## Figures and Tables

**Table 1:** TNM classification, Owen 1980 [21].

Primary Tumour Features	
T0	No evidence of tumour
TX	Tumour proven by presence of malignant cells in bronchopulmonary secretions but not seen via radiography or bronchoscopy
T1	Solitary tumour surrounded by lung or visceral pleura
T2	Multiple tumours of any size
T3	Tumour invading neighbouring tissues
Regional lymph nodes (RLN)	
N0	No evidence of RLN involvement
N1	Bronchial LN involved
N2	Distant LN involved
Distant metastasis	
M0	No evidence of distant metastasis
M1	Distant metastasis detected

**Table 2:** Classification of malignant tumours, 2017 [23].

T	Size (cm)	Solitary vs. Multiple Nodules	Organ Invasion
T1	≤3	Solitary	None
T2	>3 to ≤5	Solitary	Visceral pleura, main bronchi (not carina)
T3	>5 to ≤7	Separate nodule(s) in same lobe	Chest wall, pericardium, phrenic nerve
T4	>7	Separate nodule(s) in ipsilateral lung lobe(s)	Mediastinum, diaphragm, heart, great vessels, recurrent laryngeal, nerve, carina, trachea, oesophagus, spine
N			
N0	No lymph node metastasis		
N1	Ipsilateral tracheobronchial lymph node		
N2	Distant lymph node metastasis		
M			
M0	No distant metastasis		
M1	Malignant effusion, contralateral lung lobe metastasis, extra-thoracic metastasis		
Stage 1	T1, N0, M0		
Stage 2	T2, N0, M0; T3, N0, M0; T1–2, N1, M0		
Stage 3	T4, N0, M0; T3–4, N1, M0; T1–4, N2, M0		
Stage 4	T1–4, N1–2, M1		

**Table 3:** Study population: signalment.

**N°**	**Breed**	**Age (Years)**	**Sex**	**Weight (Kg)**
1	Bernese Mountain	8	M	37
2	Crossbreed	4	SF	4
3	American Staffordshire Terrier	13	NM	35
4	Border Collie	10	SF	21
5	Border Collie	7	M	24
6	Crossbreed	11	M	25
7	Crossbreed	10	NM	22
8	Crossbreed	10	SF	27
9	English Setter	7	NM	27
10	English Setter	8	F	28

Legend: M: male; NM: neutered male; F: female; SF: spayed female.

**Table 4:** Study population: clinical, treatment and outcome features.

N°	Clinical Signs	Pleural Effusion	Tumour Location	Total Number of VRL Administrations	VCOG Toxicity	TTP (Days)	ST (Days)
1	None	NO	Pulmonary + Hepatic Mts	8	A grade II V grade I	88	160
2	Dyspnea	YES	Diffuse Pulmonary	8 *	N grade IV V grade I	84	635
3	Weight Loss	NO	Diffuse Pulmonary	10	NO	112	120
4	Weight Loss	NO	Hepatic Mts	8	NO	90	90
5	Dyspnea	YES	Diffuse Pulmonary + Hepatic Mts	1	NV	7	7
6	Cough	NO	Diffuse Pulmonary	8 *	N grade II	84	100
7	Weight Loss	NO	Diffuse Pulmonary	8 *	NO	90	90
8	None	NO	Diffuse Pulmonary	9	N grade II	98	100
9	Dyspnea	YES	Diffuse Pulmonary	8	N grade I	84	90
10	None	NO	Diffuse Pulmonary	10	N grade I	112	120

Legend: A: anorexia; N: neutropenia; V: vomiting; TTP: time to progression; ST: survival time. *: concurrent piroxicam treatment.

## Data Availability

Raw data can be made available upon reasonable request.

## Supplementary Materials

The following supporting information can be downloaded at: https://www.mdpi.com/article/10.3390/vetsci10120664/s1, Table S1: Summary of the adverse events, previously published, in tumour-bearing dogs receiving vinorelbine.

## References

[1] Brodey R.S., Craig P.H. (1965). Primary Pulmonary Neoplasms in the Dog: A Review of 29 Cases. J. Am. Vet. Med. Assoc..

[2] Nielsen S.W., Horava A. (1960). Primary Pulmonary Tumors of the Dog. A Report of Sixteen Cases. Am. J. Vet. Res..

[3] Moulton J.E., von Tscharner C., Schneider R. (1981). Classification of Lung Carcinomas in the Dog and Cat. Vet. Pathol..

[4] Hahn F.F., Muggenburg B.A., Griffith W.C. (1996). Primary Lung Neoplasia in a Beagle Colony. Vet. Pathol..

[5] Griffey S.M., Kraegel S.A., Madewell B.R. (1998). Rapid Detection of K-Ras Gene Mutations in Canine Lung Cancer Using Single-Strand Conformational Polymorphism Analysis. Carcinogenesis.

[6] Rose R.J., Worley D.R. (2020). A Contemporary Retrospective Study of Survival in Dogs With Primary Lung Tumors: 40 Cases (2005–2017). Front. Vet. Sci..

[7] Mehlhaff C.J., Mooney S. (1985). Primary Pulmonary Neoplasia in the Dog and Cat. Vet. Clin. N. Am. Small Anim. Pract..

[8] McNiel E.A., Ogilvie G.K., Powers B.E., Hutchison J.M., Salman M.D., Withrow S.J. (1997). Evaluation of Prognostic Factors for Dogs with Primary Lung Tumors: 67 Cases (1985–1992). J. Am. Vet. Med. Assoc..

[9] Ogilvie G.K., Weigel R.M., Haschek W.M., Withrow S.J., Richardson R.C., Harvey H.J., Henderson R.A., Fowler J.D., Norris A.M., Tomlinson J. (1989). Prognostic Factors for Tumor Remission and Survival in Dogs after Surgery for Primary Lung Tumor: 76 Cases (1975–1985). J. Am. Vet. Med. Assoc..

[10] Kawabe M., Kitajima Y., Murakami M., Iwasaki R., Goto S., Sakai H., Mori T. (2019). Hypofractionated Radiotherapy in Nine Dogs with Unresectable Solitary Lung Adenocarcinoma. Vet. Radiol. Ultrasound.

[11] Ogilvie G.K., Obradovich J.E., Elmslie R.E., Vail D.M., Moore A.S., Straw R.C., Dickinson K., Cooper M.F., Withrow S.J. (1991). Efficacy of Mitoxantrone against Various Neoplasms in Dogs. J. Am. Vet. Med. Assoc..

[12] Poirier V.J., Burgess K.E., Adams W.M., Vail D.M. (2004). Toxicity, Dosage, and Efficacy of Vinorelbine (Navelbine) in Dogs with Spontaneous Neoplasia. J. Vet. Intern. Med..

[13] Rahmani R., Martin M., Barbet J., Cano J.P. (1984). Radioimmunoassay and Preliminary Pharmacokinetic Studies in Rats of 5’-Noranhydrovinblastine (Navelbine). Cancer Res..

[14] Rowinsky E.K. (2011). Chapter 13: Antimitotic Drugs. Cancer Chemotherapy and Biotherapy: Principles and Practice.

[15] Faller B.A., Pandit T.N. (2011). Safety and Efficacy of Vinorelbine in the Treatment of Non-Small Cell Lung Cancer. Clin. Med. Insights Oncol..

[16] Rowinsky E.K., Donehower R.C. (1991). The Clinical Pharmacology and Use of Antimicrotubule Agents in Cancer Chemotherapeutics. Pharmacol. Ther..

[17] Kobayashi S., Sakai T., Dalrymple P.D., Wood S.G., Chasseaud L.F. (1993). Disposition of the Novel Anticancer Agent Vinorelbine Ditartrate Following Intravenous Administration in Mice, Rats and Dogs. Arzneimittelforschung.

[18] Grant I.A., Rodriguez C.O., Kent M.S., Sfilgoi G., Gordon I., Davis G., Lord L., London C.A. (2008). A Phase II Clinical Trial of Vinorelbine in Dogs with Cutaneous Mast Cell Tumors. J. Vet. Intern. Med..

[19] Wouda R.M., Miller M.E., Chon E., Stein T.J. (2015). Clinical Effects of Vinorelbine Administration in the Management of Various Malignant Tumor Types in Dogs: 58 Cases (1997–2012). J. Am. Vet. Med. Assoc..

[20] Kaye M.E., Thamm D.H., Weishaar K., Lawrence J.A. (2015). Vinorelbine Rescue Therapy for Dogs with Primary Urinary Bladder Carcinoma. Vet. Comp. Oncol..

[21] Owen L.N. (1980). Veterinary Public Health Unit & WHO Collaborating Center for Comparative Oncology. TNM Classification of Tumours in Domestic Animals.

[22] Lee B.M., Clarke D., Watson M., Laver T. (2020). Retrospective Evaluation of a Modified Human Lung Cancer Stage Classification in Dogs with Surgically Excised Primary Pulmonary Carcinomas. Vet. Comp. Oncol..

[23] Brierley James D., Gospodarowicz May K. (2016). TNM Classification of Malignant Tumours.

[24] LeBlanc A.K., Atherton M., Bentley R.T., Boudreau C.E., Burton J.H., Curran K.M., Dow S., Giuffrida M.A., Kellihan H.B., Mason N.J. (2021). Veterinary Cooperative Oncology Group-Common Terminology Criteria for Adverse Events (VCOG-CTCAE v2) Following Investigational Therapy in Dogs and Cats. Vet. Comp. Oncol..

[25] Nguyen S.M., Thamm D.H., Vail D.M., London C.A. (2015). Response Evaluation Criteria for Solid Tumours in Dogs (v1.0): A Veterinary Cooperative Oncology Group (VCOG) Consensus Document. Vet. Comp. Oncol..

[26] Thamm D.H., Vail D.M. (2015). Veterinary Oncology Clinical Trials: Design and implementation. Vet. J..

[27] Hirschberger J., DeNicola D.B., Hermanns W., Kraft W. (1999). Sensitivity and Specificity of Cytologic Evaluation in the Diagnosis of Neoplasia in Body Fluids from Dogs and Cats. Vet. Clin. Pathol..

[28] Alleman A.R. (2003). Abdominal, Thoracic, and Pericardial Effusions. Vet. Clin. N. Am. Small Anim. Pract..

[29] Cui R., Yoshioka M., Takahashi F., Ishida H., Iwakami S., Takahashi K. (2008). Vinorelbine Is Effective for the Malignant Pleural Effusion Associated with Lung Cancer in Mice. Anticancer. Res..

[30] Depierre A., Chastang C., Quoix E., Lebeau B., Blanchon F., Paillot N., Lemarie E., Milleron B., Moro D., Clavier J. (1994). Vinorelbine versus Vinorelbine plus Cisplatin in Advanced Non-Small Cell Lung Cancer: A Randomized Trial. Ann. Oncol..

[31] Polton G., Finotello R., Sabattini S., Rossi F., Laganga P., Vasconi M.E., Barbanera A., Stiborova K., Rohrer Bley C., Marconato L. (2018). Survival Analysis of Dogs with Advanced Primary Lung Carcinoma Treated by Metronomic Cyclophosphamide, Piroxicam and Thalidomide. Vet. Comp. Oncol..

[32] Wolff H., Saukkonen K., Anttila S., Karjalainen A., Vainio H., Ristimäki A. (1998). Expression of Cyclooxygenase-2 in Human Lung Carcinoma. Cancer Res..

[33] Doré M. (2011). Cyclooxygenase-2 Expression in Animal Cancers. Vet. Pathol..

[34] Khan K.N., Knapp D.W., Denicola D.B., Harris R.K. (2000). Expression of Cyclooxygenase-2 in Transitional Cell Carcinoma of the Urinary Bladder in Dogs. Am. J. Vet. Res..

[35] Spugnini E.P., Porrello A., Citro G., Baldi A. (2005). COX-2 Overexpression in Canine Tumors: Potential Therapeutic Targets in Oncology. Histol. Histopathol..

[36] Knapp D.W., Richardson R.C., Chan T.C., Bottoms G.D., Widmer W.R., DeNicola D.B., Teclaw R., Bonney P.L., Kuczek T. (1994). Piroxicam Therapy in 34 Dogs with Transitional Cell Carcinoma of the Urinary Bladder. J. Vet. Intern. Med..

