# Supplementary material for: Vinorelbine as First-Line Treatment in Stage IV Canine Primary Pulmonary Carcinoma
Source: Vet Sci. 2023 Nov 22;10(12):664. doi: 10.3390/vetsci10120664 (PMC10747721; doi:10.3390/vetsci10120664)
Supplement: Supplementary file 1 [file vetsci-10-00664-s001.zip › vetsci-2705531-supplementary.pdf]

Table S1: Summary of the adverse events, previously published, in tumour bearing dogs receiving vinorelbine

Reference Number	Years of Pubblication	Authors	N. Dogs	Tumour	AE
19	2015	Wouda et al.	58	Various	N4(6); N3(5); N2(8); N1(4); T2 (3); T1(1).
20	2013	Kaye et al.	14	Transitional Cells Carcinoma	N1(11); N2(1); N3(3); N4(2); A1(1); A2(1); T1(1); T3(1); V1(6); V2(1); D1(2); D2(3); D3(1); Ax1(3); Ax3(1).
18	2008	Grant et al.	24	Mast Cell Tumour	N1(4); N3 (6); N4(3); Ax1 (3); Ax2 (1); A3(1); D1(2); D3 (1); V1(5); V3(1).
12	2004	Poirier	19	Various	N2(4); N4(4).

Abbreviations: N: neutropenia; T: trombocythopenia; A: anaemia; V: vomiting; Ax: anorexia; D: diarrhoea. Toxicity grading in according to VCOG (24)
